# Analysis of seasonal changes in vegetation cover in the La Caimanera marsh (Colombia) using remote sensing and satellite sensor imagery

**DOI:** 10.12688/f1000research.164770.2

**Published:** 2026-01-02

**Authors:** Carlos Segundo Cohen-Manrique, Jhonatan Andrés Rodríguez-Manrique, Andrea Fernanda Burbano-Bustos, Javier Andrés Castro-Rodríguez

**Affiliations:** 1 Faculty of Engineering, Universidad Tecnologica de Bolivar, Cartagena, Bolívar, Colombia; 2 Faculty of Basic Sciences, Engineering and Architecture, Corporación Universitaria del Caribe, Sincelejo, Sucre, Colombia

**Keywords:** Mangroves, remote sensing, LULC, data analysis

## Abstract

Mangroves are ecosystems that link freshwater, land, and oceans. They are also home to vast biodiversity of species and provide vital resources to many coastal communities worldwide. However, in various parts of the world, these natural ecosystems have been subjected to anthropogenic actions that compromise their subsistence. Therefore, the aim of this study was to analyse seasonal changes in vegetation cover in the La Caimanera marsh (Colombia) using remote sensing and satellite sensor imagery. Methodologically, machine learning techniques were used, including semi-supervised and supervised learning, using Landsat, Copernicus, and Planet images. The influence of mangrove vegetation cover on pollutants was explored using remote sensing data and in-situ measurements. The results obtained indicated that in 2015 and 2024, the ecosystem showed stability, with slight reductions in mangrove cover and water bodies. In contrast, a slight increase in urbanized areas was also evident. The NDWI, MNDWI, OC3, and QAA spectral indices were used to monitor water dynamics and water quality. The results reflected stability in water conditions between 2010 and 2020, with a slight reduction in 2023. The increase in chlorophyll-a and reduction in turbidity in 2023 showed alterations in water quality. In addition, the predominance of mature mangrove trees, which comprised 83% of the vegetation, is noteworthy, reflecting a healthy and stable ecosystem. The greater density and homogeneity of the mangrove canopy observed in this study suggests a positive ecosystem response and greater resilience to past environmental changes. It is concluded that the changes experienced by the La Caimanera marsh reflect a balance between conservation and development, highlighting the effectiveness of territorial management policies implemented to maintain the ecosystem’s resilience in the face of human pressures.

## Introduction

Mangroves are critical ecosystems that connect the freshwater, land, and sea. They harbor great diversity and provide protection and resources to countless coastal communities worldwide. The total extent of mangrove habitats globally covers approximately 148,000 km ^2^ and is mostly distributed along the coasts of 120 countries. ^1^ This was equivalent to approximately 15% of the global coastline. ^2^ For several decades, these areas have been the subject of international study and protection for what they represent as natural reservoirs. Mangroves not only harbor rich marine and plant biodiversity but also contain diverse physicochemical components that contribute to the ecological stability of coastal zones and surrounding regions. ^3^ Mangrove reservoirs act as highly reliable natural barriers to prevent extreme weather changes and storm surges, protecting coastal populations from extreme disasters, such as storm surges, hurricanes, and tsunamis. In addition, they are a vital source for fish, mollusks, birds, human settlements, and other species, as they are often used as nurseries for fish and to provide valuable natural resources in areas such as medicine, forestry (biomass), and the stabilization of coastal sediments through their roots. These ecosystems are often attractive tourist destinations that contribute to strengthening local economies. ^4, 5^

Mangroves are terrestrial carbon reservoirs that play a crucial role in the transfer of dissolved organic carbon to the ocean. This type of coastal wetland dominated by woody plants is found mainly along Earth’s intertropical zone, which encompasses the region located between the Tropics of Cancer and Capricorn. ^6^ The ecological and biological functions of mangroves have been a highly relevant research focus over the years because of their key role in climate change mitigation and flood protection. ^7^ In addition, their biodiversity and role as global temperature regulators have been considerably deepened. ^8, 9^ However, these ecosystems face various anthropogenic pressures such as deforestation, hydraulic alteration of coastal systems, urban expansion, charcoal production, and pollution, which increase their risk of subsistence. ^10^ Regarding the impact of land cover variation on aquatic ecosystems, the literature is extensive and diverse, especially in the use of satellite imagery and field data. Locke ^11^ offered a critical view on the impacts of land use and land cover (LULC) on water quality, noting that activities such as agriculture, urban development, mining and commercial forestry are sources of diffuse pollution. In parallel, Saoum and Sarkar ^12^ analyzed the dynamics of forest cover change in Sundarbans, Bangladesh, using remote sensing and time series, highlighting an alarming annual reduction in mangrove area, associated with environmental factors such as precipitation and surface temperature.

This study also linked the decline in mangrove area to the intensification of human activities, such as fishing and logging, highlighting the urgency of implementing proactive conservation strategies. They also highlighted the delayed effects of deforestation, which compromised the resilience of the ecosystem and aggravated environmental deterioration in the long term. On the other hand, Avtar ^13^ complemented the analysis of satellite images with social surveys in their study on changes in mangrove ecosystems. In the Ba and Rewa River delta (Fiji), it was identified that approximately 50% of the local population depends directly on the resources provided by mangroves, using the area daily for food and other essential products, highlighting the link between human use and the sustainability of these ecosystems.

In the field of mangrove research using remote sensing, satellite imagery, and sensors, Maurya ^14^ analyzed various remote sensing techniques applied to mapping and monitoring mangrove ecosystems, highlighting both their capabilities and limitations. In their study, digital image classification techniques were compared in terms of the spectral and spatial resolutions of the images used to evaluate the classification accuracy for obtaining specific information on different classes of mangroves. Yu ^15^ used a time series of Landsat satellite images covering 1990 to 2020 to monitor changes in mangrove cover and extent on Hainan Island, China. These researchers used the standard deviation ellipse method, examined the spatial distribution of mangroves, and applied the CA-Markov model to project possible changes in the extent of mangroves by 2025 and 2030. In another study, Mondal ^16^ assessed the intra-annual and inter-annual coastal water quality in the estuarine system of Sundarbans through remote sensing and in situ measurements. In this study, they used Landsat 8-OLI sensor data and water quality parameters, such as temperature, pH, dissolved oxygen, phosphate, and nitrate, and employed advanced machine learning algorithms, such as Random Forest (RF), K-Nearest Neighbor (KNN), Support Vector Machine (SVM), and Artificial Neural Network (ANN), to predict critical water quality indicators in this region. In Colombia, mangroves cover approximately 380,000 ha, of which approximately 77% are located on the Pacific coast and 23% on the Caribbean coast. ^17^

Under the framework of the National Mangrove Management Plan (2002), these areas are managed according to international guidelines to guarantee the sustainability of these critical ecosystems. The plan promotes the rational use of resources, early detection of water stress, and biodiversity conservation, along with climate regulation and carbon sequestration, which are all essential factors for their protection and for the well-being of local communities. ^18^ However, deforestation of mangrove ecosystems in Colombia has also occurred over time. Since 1960, approximately 57% of mangrove cover has been lost owing to pollution, climate change, erosion, and the lack of conservation policies for this ecosystem. Therefore, it is necessary to investigate the evolution of mangroves in Colombian territory to facilitate decision-making from the academy to the state.

This study is particularly significant because the La Caimanera marsh (Sucre, Colombia) has not previously been analyzed in terms of how variations in vegetation cover influence pollution dynamics. This area holds major importance for the region, as it is primarily a fishing and tourism zone that supports a large part of the local economy. However, in recent years, this ecosystem has faced growing challenges related to pollution and progressive loss of vegetation cover, which underscores the urgent need to generate scientific evidence to support sustainable management strategies. Understanding the interaction between vegetation cover and pollution levels is essential for designing restoration and conservation plans that safeguard both biodiversity and the livelihoods of the surrounding communities. Furthermore, by integrating satellite imagery, in situ sensor data, and machine learning approaches, this research contributes to the development of cost-effective and replicable monitoring frameworks applicable to other tropical wetlands under similar anthropogenic pressures. This mangrove ecosystem constitutes not only a critical habitat for regional biodiversity but also a direct source of livelihood for local communities that depend on fishing and tourism. Analysing the relationship between vegetation cover variation and pollution levels makes it possible to understand the processes that threaten ecological balance while providing scientific evidence to strengthen conservation and sustainable land management strategies.

The importance of this study lies in the integration of emerging technologies, such as remote sensing and machine learning, applied to the environmental monitoring of tropical ecosystems. The combined use of multitemporal satellite imagery and in situ sensor data allows the establishment of quantitative relationships between vegetation cover dynamics and water quality, offering a low-cost and replicable approach for the environmental management of coastal wetlands in Colombia and other Caribbean regions. This approach provides a cost-effective and scalable monitoring framework for tropical wetlands under similar pressures.

On the other hand, this study is particularly relevant because its results can support the formulation of public policies aimed at the integrated management of coastal ecosystems in the Colombian Caribbean. The evidence generated contributes to data-driven decision-making, the monitoring of the Sustainable Development Goals (SDGs 13, 14, and 15), and the strengthening of institutional strategies for mangrove conservation in the face of urban development pressures and climate change.

Therefore, in the particular case of the La Caimanera marsh, to the best of our knowledge, no scientific studies have analyzed the impact of vegetation cover variation on pollution levels in this ecosystem. Considering that this mangrove scheme represents the livelihood of numerous local families, it is a source of biodiversity in the region and boosts the tourism sector of the Department of Sucre, Colombia. Therefore, to analyse the evolution of this ecosystem, this study analysed the way in which vegetation cover can affect pollution levels in the area surrounding the La Caimanera marsh (Colombia), implementing machine learning techniques using semi-supervised and supervised learning with Landsat, Copernicus, and Planet images. Thus, the relationship between vegetation cover and pollutants can be evaluated using remote sensing and in situ sensor data. Additionally, the behaviour of the time series of the normalised difference vegetation index (NDVI), normalised difference water index (NDWI), and Land Surface Temperature (LST) in the ROI was evaluated using MODIS data extracted from the Google Earth Engine platform. Therefore, the aim of this study was to analyse seasonal changes in vegetation cover in the La Caimanera marsh (Colombia) using remote sensing and satellite sensor imagery.

## Materials and methods

### Study area

The La Caimanera marsh is situated in the Gulf of Morrosquillo subregion, in the northern part of the Department of Sucre (Colombia). The coordinates of the La Caimanera marsh correspond to 9°25’57.42” latitude and 75°37’53.64” west W.

La Caimanera marsh is located in the coastal zone of the Gulf of Morrosquillo, in the Department of Sucre, Colombia, approximately 9 km from the municipal capital of Santiago de Tolú. The wetland covers an estimated surface area of 192 ha and receives hydrological input from the Escobalito, San Antonio, and La Lata streams, maintaining an estuarine connection with the Caribbean Sea through a natural channel. This connection supports a highly productive and dynamic coastal ecosystem.

The study area is characterized by a heterogeneous landscape, including 184 hectares of swamp zone dominated by mangrove species such as Avicennia germinans, Laguncularia racemosa, and Rhizophora mangle, along with extensive virgin mangrove areas. These comprise a 559-hectare sector currently under ecological recovery and an 865-hectare undisturbed forest core that remains difficult to access. The site is bordered by the Caribbean Sea to the north, private livestock farms to the east and south, and human settlements with intensive tourism to the west. ^19^

From a geophysical standpoint, the area presents a xerophytic savanna climate, with an annual mean temperature of approximately 27 °C and average precipitation near 1,200 mm. ^20^ The hydrological balance shows a deficit during the dry season (December to February) and a significant recharge during the rainy months. Soils are predominantly sandy and saline, with low fertility, while mangrove soils are waterlogged and highly conservative. ^21^ The coastal alluvial plain supports two life zones: premontane dry forest to the north and tropical dry forest to the south.

Socio-demographically, the municipality of Coveñas—where La Caimanera marsh is located—has undergone marked growth over the past decade. Its population has increased from approximately 17,000 inhabitants in 2010 to over 19,500 by 2023, with projections approaching 20,000 by 2025. The local economy is primarily based on fishing, tourism, and port-related activities complemented by emerging initiatives in the blue economy. Despite progress in infrastructure and public services, challenges persist in terms of housing quality, Internet access, and social equity. The area has a strong presence of Afro-Colombian communities and a predominantly young demographic structure. ^22^ The balance between economic expansion and ecosystem conservation remains a central challenge for sustainable development in this coastal region.

The La Caimanera marsh and its mangrove ecosystem are currently exposed to multiple sources of anthropogenic pollution. The main contaminants identified in the area include plastic residues, pesticides, industrial discharges, solid waste, and agricultural fertilisers, which enter wetlands through surface runoff and local drainage channels. Recent environmental monitoring and field surveys have also reported increases in harmful algal blooms, coloured dissolved organic matter (CDOM), suspended solids, sediments, and general waste accumulation. These factors contribute to the degradation of water quality and directly affect the ecological balance of the mangrove system, emphasizing the need to understand how vegetation cover variation interacts with pollution dynamics in this coastal ecosystem. ^18^^,^^23^

**Figure 1.**
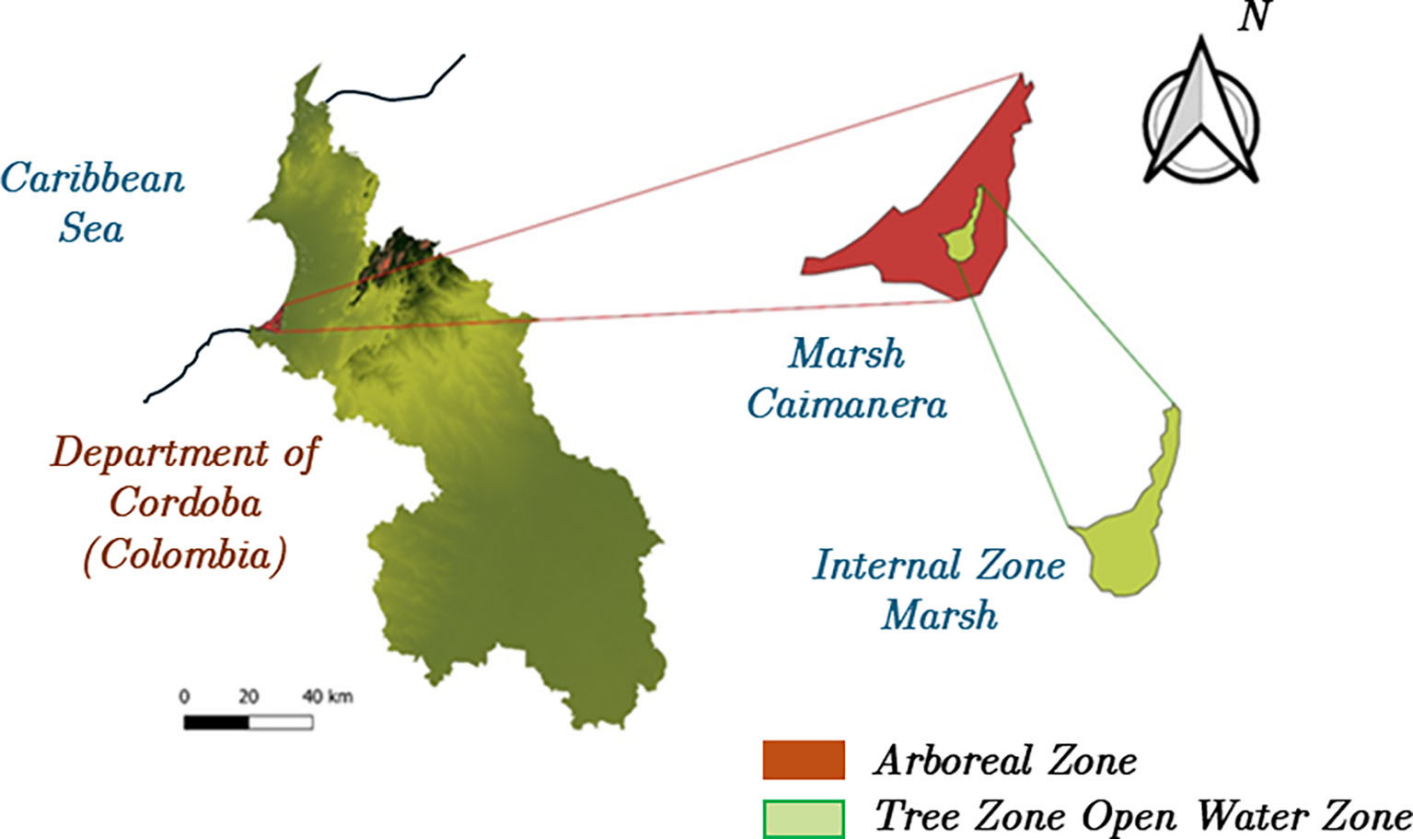
Geographic location of the La Caimanera marsh (Colombia).

Figure 1 illustrates the zoning of the La Caimanera marsh in the two visually contrasting areas. The first corresponds to the area covered by mangroves (in red), predominantly composed of at least four different mangrove species that form a dense tree canopy at medium to high altitudes, creating a closed forest landscape characteristic of these ecosystems. ^24^ In the centre of the swamp, there is a section of open water that lacks surface vegetation, which makes it a favourable area for tourism, recreation, and artisanal fishing. The La Caimanera marsh is bordered to the north by the Atlantic Ocean, where it is connected by a natural channel that plays a fundamental role in the hydrological and ecological dynamics of the system. This channel facilitates the exchange of water between the marsh and sea, directly influencing critical parameters such as salinity, water quality, and the stability of the mangrove ecosystem, promoting the health and resilience of these key habitats for the region.

### Source of data

Data were obtained from various satellite sources. Their high value in the analysis of mangrove ecosystems using remote sensing is highlighted. In this study, different satellite and geospatial datasets were combined to capture complementary spatial and temporal information. PlanetScope imagery (4.7 m resolution) was used for high-precision monitoring of vegetation cover and small-scale land use changes. Landsat 7, 8, and 9 data (30 m resolution) provided long-term spectral information to assess vegetation indices and detect trends in mangrove dynamics. Moderate Resolution Imaging Spectroradiometer (MODIS) products (250–500 m resolution) were employed for time-series analyses of the vegetation indices (NDVI and NDWI) ^25^ and land surface temperature (LST), allowing the assessment of seasonal and inter-annual variability. Sentinel-2 images from the Dynamic World platform (10 m resolution) were used to perform land use/land cover (LULC) classification and to map the mangrove extent with greater spatial detail. ^26^ Finally, the Global Mangrove Watch (GMW) dataset (JAXA and Aberystwyth University) provided validated annual estimates of mangrove areas from 1996 to 2020, enabling cross-validation of the classification results and long-term change detection. Together, these sources offered a multi-scale perspective that strengthened the robustness and reproducibility of the environmental analysis.

Images from the Planet Application Program Interface ( https://www.planet.com/) were used, with a spatial resolution of 4.7 meters and blue (B), green (G), red (R), and near-infrared (NIR) spectral bands. In addition, images from Landsat 7, 8, and 9 satellites ( https://landsat.gsfc.nasa.gov/) were included, which provide resolutions of up to 30 m, which is ideal for medium-scale environmental monitoring. For time series and advanced analyses, MODIS collection data available from Earth Engine were used, such as MOD44B.006 Terra Vegetation Continuous Fields Yearly Global, with a resolution of 250 m per pixel, and MCD12Q1.061 MODIS Land Cover Type Yearly Global, with a resolution of 500 m. The MOD11A2 V6.1 product was used for land surface temperature (LST) estimation.

**Figure 2.**
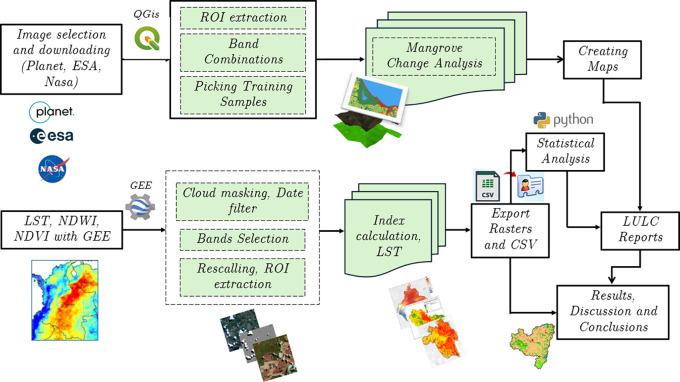
Methodological framework used for image acquisition and processing.

For the analysis of land use and land cover, Sentinel images from the Dynamic World platform, which manages a global scale with a resolution of 10 m ( https://dynamicworld.app/), were accessed. To evaluate mangrove dynamics and their evolution, data from the Global Mangrove Watch (GMW) ( https://www.eorc.jaxa.jp/ALOS/en/dataset/gm_e.htm), developed in collaboration with the Japan Aerospace Exploration Agency (JAXA) and Aberystwyth University, were used, which provided accurate estimates of the extent and changes in mangrove forests at 11 annual epochs between 1996 and 2020 ( https://www.globalmangrovewatch.org/). In addition, NASA Landsat SEDAC Global Mangrove Forest Distribution data at 30-meter resolution were used to provide a detailed analysis of spatial and temporal patterns of mangrove cover. Data were obtained from the European Union’s Global Surface Water Explorer platform ( https://global-surface-water.appspot.com/). Figure 2 illustrates the methodology used for the acquisition and processing of the satellite images used in this study.

However, the Random Forest algorithm was used, which is often preferred for its ability to generate robust and accurate results. ^27^^,^^28^ This machine learning model uses the combined output of several decision trees trained using the CART (Classification and Regression Tree) algorithm, allowing the creation of subsets of data that optimize land cover classification. ^29^ The Random Forest mathematical model is detailed in the following equation:

$$H\left(x\right)=\arg\max_{c\in C}\sum_{i=1}^{T}\;I\left(h_{i}\left(x\right)=c\right)$$

Where, T is the total number of trees in the forest, $h_{i}\left(x\right)$ is the prediction of tree i for input x , H(x) is the final prediction, I is the indicator function that becomes 1 if the prediction of tree i is class c and 0 otherwise. The following equation was used as the regression model for the Random Forest.

$$H\left(x\right)=\frac{1}{T}\sum_{i=1}^{T}\;h_{i}\left(x\right)$$

As correlations between land surface temperature (LST) and the Normalized Difference Vegetation Index (NDVI) were employed, it was necessary to ensure that both products had the same spatial resolution. To this end, NDVI, as a time series, was also extracted from the MODIS data. Specifically, the Terra MODIS MOD13Q1 product, extracted using the Google Earth Engine, was used ( Figure 2). The NDVI and LST values for each year were adjusted to the 90th percentile to minimize the influence of pixels affected by atmospheric variations. NDVI is a widely recognized index that allows the quantification of vegetation health and density. ^30^

The NDVI was derived from the MODIS MOD13Q1 product, which provides 16-day composite imagery with a 250 m spatial resolution. The index was calculated using the standard formula:

$$\text{NDVI}=\frac{\mathrm{NIR}-\mathrm{RED}}{\mathrm{NIR}+\mathrm{RED}}$$

where NIR and RED correspond to near-infrared and red reflectance bands, respectively. LST values were obtained from the MODIS MOD11A2 product, which offers 8-day composites with a 1 km spatial resolution, providing average daytime land surface temperature. Both datasets were accessed and processed using Google Earth Engine (GEE) for the period 2010–2023. Temporal aggregation was performed using annual means to reduce short-term variability. Radiometric calibration and atmospheric correction were automatically performed using MODIS preprocessing routines. These indices were selected due to their proven reliability for monitoring vegetation dynamics and surface temperature interactions in tropical mangrove ecosystems.

The methodological workflow used in this study is illustrated in Figure 2. It outlines the complete sequence of procedures applied, including satellite image selection (Planet, ESA, NASA), preprocessing and index computation in the Google Earth Engine (GEE), extraction of regions of interest (ROIs), land cover classification in QGIS using the Random Forest algorithm, and subsequent statistical analyses performed in Python. ^31^ The final outputs were used to generate land use and land cover (LULC) maps and to interpret the relationships between vegetation cover and pollution indicators.

The integration of MODIS data is essential for analyzing the temporal dynamics of vegetation health and thermal fluctuations, compensating for the temporal and spectral limitations of Landsat imagery. This approach allows filling gaps in the Landsat time series, providing a more complete long-term analysis. LST and NDVI results extracted from the Google Earth Engine were exported in raster format and processed in QGIS 3.38.2’Grenoble’ to generate detailed maps. Prior to image analysis, all satellite scenes were preprocessed to correct atmospheric and radiometric distortions using the surface reflectance products provided by each platform. Cloud and shadow masking were performed using the QA band available in Landsat and Sentinel products, and images were resampled to a uniform spatial resolution of 30 m to ensure consistency among the datasets. ^32^ This study employed a supervised classification approach with 100 training samples per class, which were selected based on visual interpretation and prior field knowledge. For each class (dense mangrove, sparse mangrove, bare land, built-up area, and waterbody), Regions of interest (ROIs) were delineated to train the Random Forest (RF) classifier implemented in the Google Earth Engine environment for each class (dense mangrove, sparse mangrove, bare land, built-up area, and water body). The RF model consisted of 150 decision trees with a maximum depth of 20 and used the Gini impurity criterion for node splitting. The model accuracy was evaluated using a confusion matrix and cross-validation procedure, achieving an overall classification accuracy of 91.3% and a kappa coefficient of 0.88. The resulting classified maps were exported and post-processed in QGIS 3.38.2 “Grenoble” for visualisation and integration with field data collected through low-cost IoT sensors. Correlation analyses between the Normalized Difference Vegetation Index (NDVI) and Land Surface Temperature (LST) were conducted in Python (version 3.12), using Pearson’s coefficient (r) to assess linear relationships between vegetation cover and surface temperature dynamics across the study area. ^33^ For the analysis, 450 points were generated within the interior areas of the bog, avoiding aquatic areas to mitigate the effect of water on the correlation between NDVI and LST. The extracted values were used in a correlation analysis performed in Python (version 3.12) to study the relationship between land surface temperature and vegetation health in the study area.

### Data analysis

The data analysis in this study was carried out using different tools, depending on the type of information and its specific purpose. QGIS 3.38.2 ‘Grenoble’ and Google Earth Engine (GEE) were used to process images from Planet, Landsat, MODIS and Sentinel satellites ( Figure 2). In particular, Landsat images were processed using QGIS, while GEE was used to handle Dynamic World data, vegetation indices such as NDVI and NDWI, land surface temperature (LST) obtained from MODIS, and water quality indices such as OC3 and QAA quality. The region of interest (ROI) was delimited by creating a mosaic of marsh images, with a focus on land cover variation, specifically mangrove areas. Major water bodies within the study boundaries were excluded from a more focused vegetation analysis. For land cover classification, a supervised classification process was conducted using JavaScript in the GEE. One hundred training samples were collected for each land-cover class based on visual interpretation and the researchers’ prior knowledge of the region. These samples allowed the surface to be categorized into five classes: dense mangroves, sparse mangroves, bare land, built-up areas, and waterbodies. For the classification algorithm, Random Forest (RF) with a set of 150 trees was used because of its ability to increase the classification accuracy by combining the results of multiple decision trees. ^27, 34^

## Results and discussion

### Characteristics of the study area

The La Caimanera marsh is distinguished by its highly diverse habitats, including beaches, mangroves, mudflats, and wetlands, making it a crucial ecosystem for the development of a wide range of animal species at different trophic levels. ^35^ These habitats harbor a wide variety of life forms, from autotrophic bacteria that decompose organic matter to aquatic mammals, including diverse invertebrates such as annelids, mollusks, and arthropods, as well as vertebrates such as fish, amphibians, reptiles, birds, and terrestrial mammals, many of which depend on mangroves to complete the fundamental stages of their life cycle. ^36^ The mangrove structure in this area is characterized by a fringe-type mangrove swamp approximately 500 m wide, starting from the main channel in the Boca de la Ciénaga sector, coinciding with that reported by the Corporación Autónoma Regional de Sucre. ^18^ In the first strip, the mangrove is predominantly monospecific and dominated by the red mangrove ( *Rhizophora mangle*). As one moves inland, the ecosystem becomes more heterogeneous, with co-dominance of black mangroves ( *Avicennia germinans*) and white mangroves ( *Laguncularia racemosa*). After this zone, the forest adopts a basin-type structure, with flooded areas during the rainy season fed mainly by the Petalaca and San Antonio streams. Black mangroves predominate here, accompanied by white mangroves, and to a lesser extent, red mangroves. ^17^

### Evaluation of indexes in the marsh area

**Figure 3.**
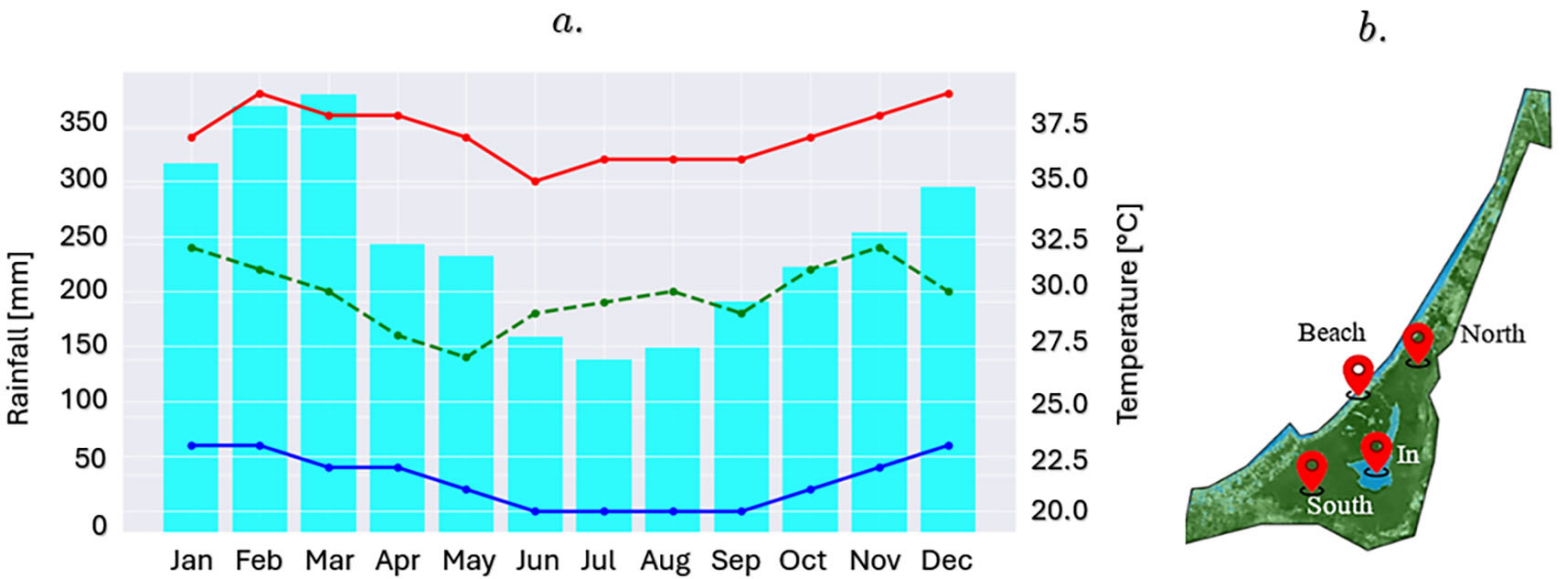
a. Climogram of the La Caimanera marsh (mean value for the period 2018-2023); b. Location of sample points for the indices.

Figure 3 shows a climogram of the maximum, minimum, and average temperatures in the study area over the last 5 years. These data were obtained from the MODIS Land Surface Temperature and Emissity (MOD11) satellite and data taken directly by researchers in the study area using low-cost sensors, such as temperature (DS18B20), solar radiation (TSL2591), and air quality (MQ-135) sensors connected to an ESP32 S3 controller to validate the satellite information obtained. This information is important for identifying the climatic patterns of the study area and the times of the year when the dry and rainy seasons occur. The Google Earth Engine (GEE) platform and satellite images from the European Space Agency (ESA) were used to generate a time series of NDVI and NDWI indices in four critical areas of the marsh. The first area, located in the southern sector, was characterized by a high density of vegetation and abundant mangroves, represented by the NDVI_south index. In contrast, the northern sector, which connects the swamp with the sea and acts as a tourist channel for residents and visitors, was monitored using the NDVI_North index. Additionally, a point was identified in the transition zone between the beach and the swamp, captured in the NDVI_beach index, while the last area of interest was located in the interior of the swamp, characterized by open waters and scarce presence of mangroves, reflected in the NDVI_swamp. The NDWI index was used in similar areas of the marsh ( Figure 3b).

**Figure 4.**
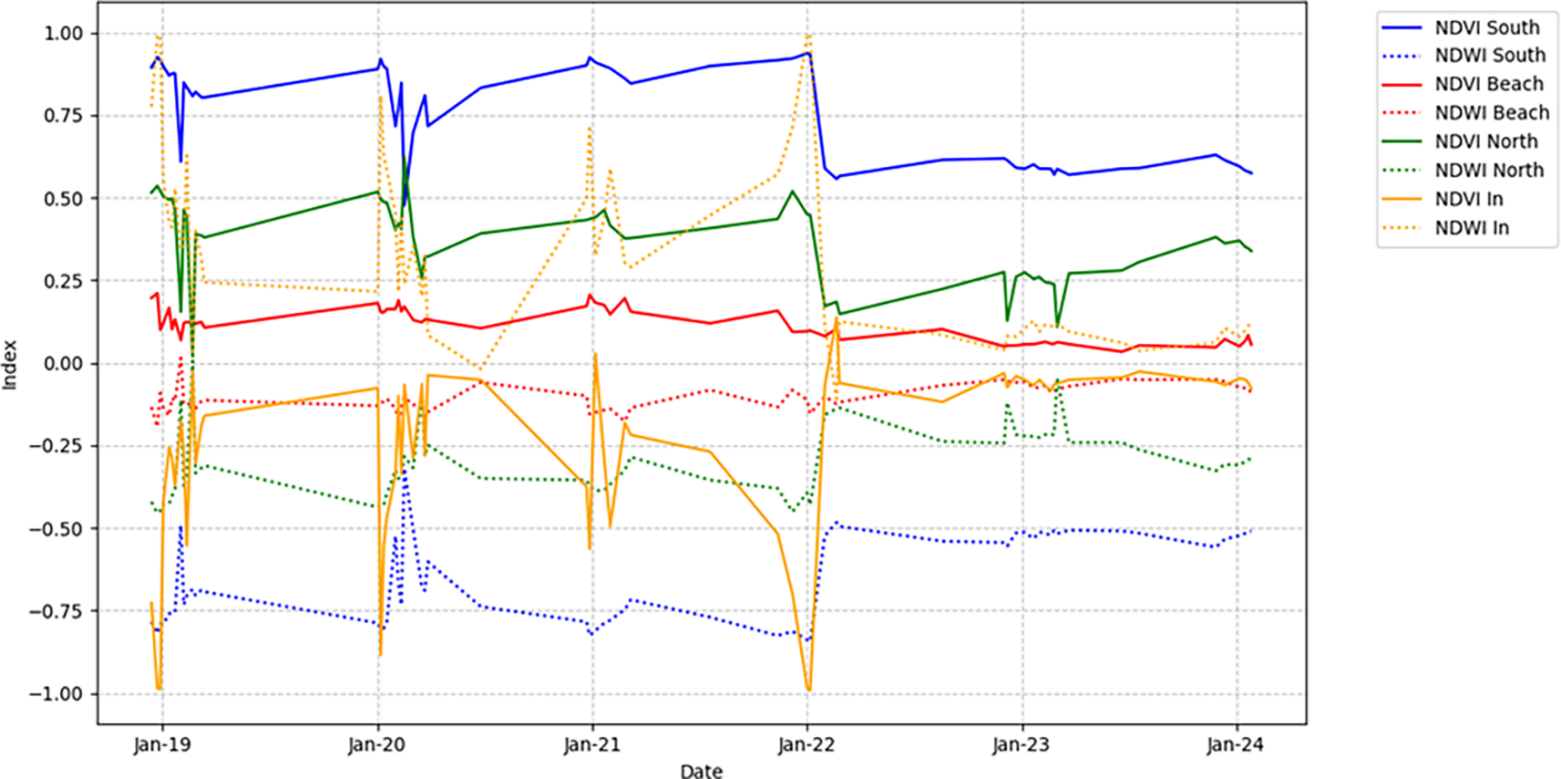
NDVI and NDWI index comparisons in four points of the La Caimanera marsh.

The values shown in Figure 4 with the yellow line correspond to NDVI_In, which represents the inner part of the marsh, characterized by open water and the scarce presence of nearby mangroves. Finally, NDVI_beach refers to the area of the marsh bordering the beach and surrounding communities.

Figure 4 presents the NDVI variation in the four zones of the La Caimanera marsh from January 2019 to January 2024, highlighting the patterns in vegetation cover. The NDVI_south (blue) shows high and stable values, reflecting dense and consistent vegetation. In contrast, NDVI_Beach (red) shows low and fluctuating values, indicating sparse vegetation, because it is a construction zone, roads, little vegetation, and high salinity. NDVI_North (green) exhibits moderate vegetation, with significant mangrove density, lower than that of the southern zone, with seasonal variations or human influences. NDVI_In (orange) shows high variability but with a tendency toward negative values because the zone is mostly unvegetated. In parallel, NDWI in Figure 4 (dotted lines) shows the humidity in each zone. The southern region (blue line) presents high negative values because the high density of mangroves prevents the detection of water by the satellite. The beach (red line) fluctuates near zero, indicating the low humidity levels characteristic of this zone. In the north (green line), the predominantly negative values throughout the period reflect moderate humidity. In contrast, the interior zone of the marsh (orange line) shows high peaks associated with the presence of open water during most of the year.

**Figure 5.**
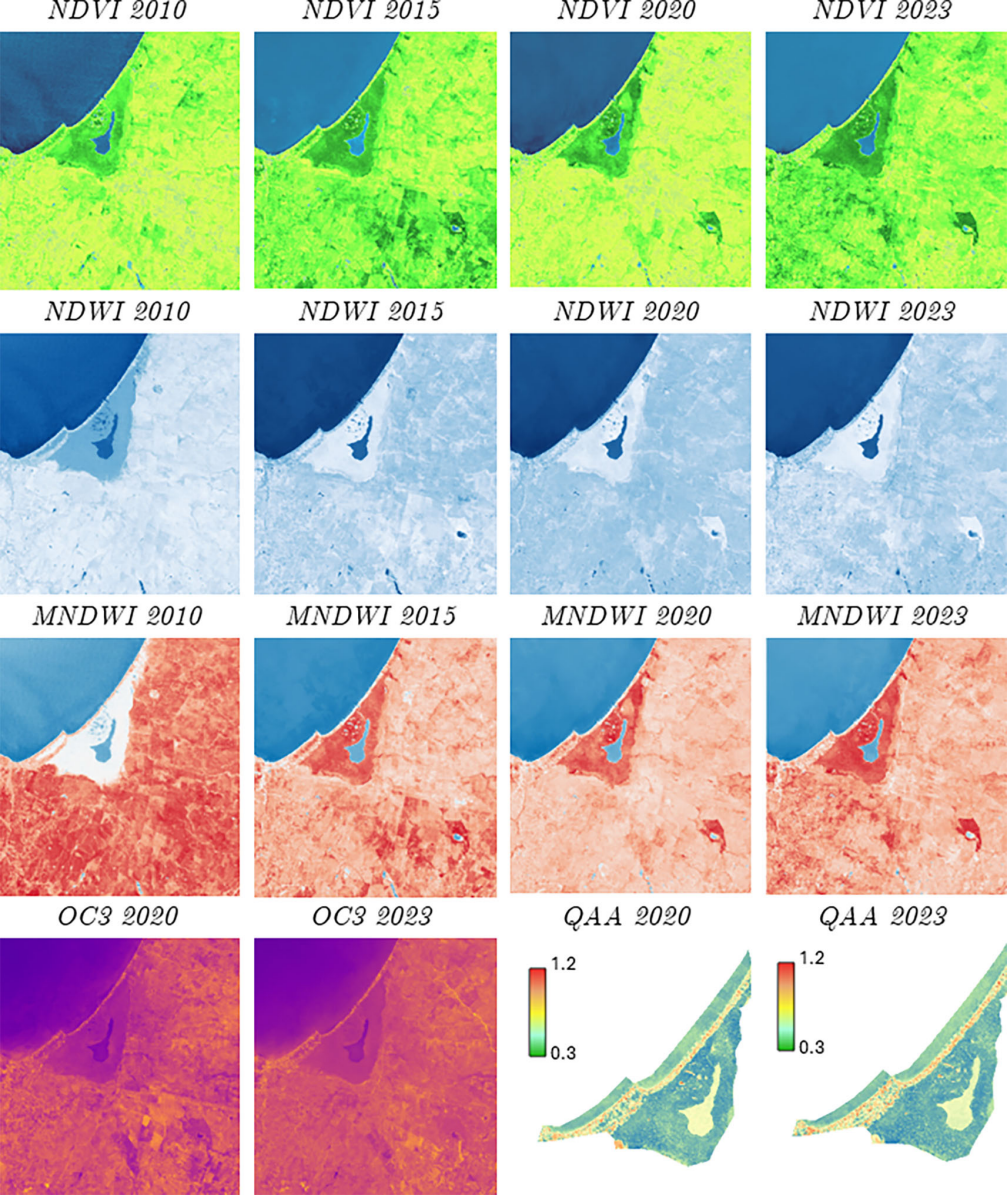
Satellite evaluation of the area of interest using NDVI, NDWI, MNDWI and OC3 and QAA quality parameters.

Figure 5 presents various indices applied to the study area, including OC3 and QAA, which are fundamental for estimating the chlorophyll concentration and, therefore, to assess the health of phytoplankton in the ROI. In particular, the Normalized Difference Vegetation Index (NDVI) in the La Caimanera marsh, Colombia, was analyzed for July 2010, 2015, 2020, and 2023, revealing significant dynamics in both vegetation and water bodies in the area.

The NDVI is a key tool in remote sensing and is used to measure the density and health of vegetation, providing an accurate representation of the spatial distribution of ecosystems. This temporal analysis allowed the identification of crucial variations in vegetation patterns and changes in the extent of the main water body, providing essential information for environmental management and ecosystem conservation. In 2010, most of the terrestrial area showed moderately dense vegetation (light green), with some areas of mangroves surrounding the inner water body of the marsh, and very dense vegetation (dark green). The main water body, represented in blue, is clearly visible, whereas a small interior water body, corresponding to the marsh, is distinguished by lighter tones. By 2015, a significant increase in dense vegetation was observed, particularly in the mangrove zone in the center-south of the study area. The marsh appears to have slightly increased in size, which could be related to changes in water levels or an increase in the surrounding vegetation. Despite this growth, moderately dense vegetation continued to predominate in areas farther from the coast. In 2020, there was a general decrease in dense vegetation, mostly replaced by moderately dense or sparse vegetation (light green to greenish-yellow). The inland water body maintained its size, but the change in vegetation density suggests possible variations in environmental conditions or land management during this period. Finally, in 2023, a partial recovery of dense vegetation was detected, although it did not reach the levels observed in 2015. The landscape appears more heterogeneous, with a mixture of areas of dense and moderate vegetation, reflecting greater variability in the ecosystem conditions. The marsh, on the other hand, appears to have been slightly reduced in extent, which could be due to variations in water levels or changes in riparian vegetation.

Regarding the NDWI, for the years 2010 to 2015, there were slight increases in the intensity of blue in the marsh, which could indicate an increase in water content or an improvement in water quality. Similarly, for the time window from 2015 to 2020, the coloration remains similar, suggesting a certain stability in the water conditions; for the period from 2020 to 2023, a slight decrease in the intensity of blue in the marsh is clearly observed, which would be related to a reduction in the volume of water or changes in water quality. However, the changes in the NDWI and MNDWI index colors for the area around the bog show lighter shades of blue, which change subtly over the years. This indicates variations in the soil moisture or surrounding vegetation. Finally, the evaluation of both indices showed some darker spots scattered in the area, which represent small bodies of water (jabueyes) or areas of higher humidity. These values appear to have remained relatively constant over the years. Regarding water quality in the marsh, the OC3 and Quasi-Analytical Algorithm (QAA) parameters were used for 2020 and 2023, generating the following information: the OC3 algorithm is commonly used to estimate chlorophyll-a concentration in oceanic and coastal waters, and the images show orange and purple tones as predominant colors with a slight intensification of purple color in 2023 compared to 2020, especially in the marsh area and some terrestrial areas. This indicates a small increase in the chlorophyll-a concentration or changes in the optical properties of the surrounding water and land.

The QAA algorithm was used to derive the inherent optical properties of water such as light absorption and scattering. The predominant colors were green, blue, and yellow tones, with the bog having a lighter blue-green tone. Figure 5 shows a slight reduction in the intensity of the blue-green color in the marsh in 2023 compared to 2020, indicating a lower amount of sediment and less turbidity in the water. Finally, the images were downloaded using the Copernicus Browser platform service of the European Space Agency, using a cloudiness filter of 10% allowed, with high-resolution visualization, WSG 84 (EPSG 4326) coordinate system using the Senttinel-2 L2A satellite sensor.

### Variations in vegetation cover

**Figure 6.**
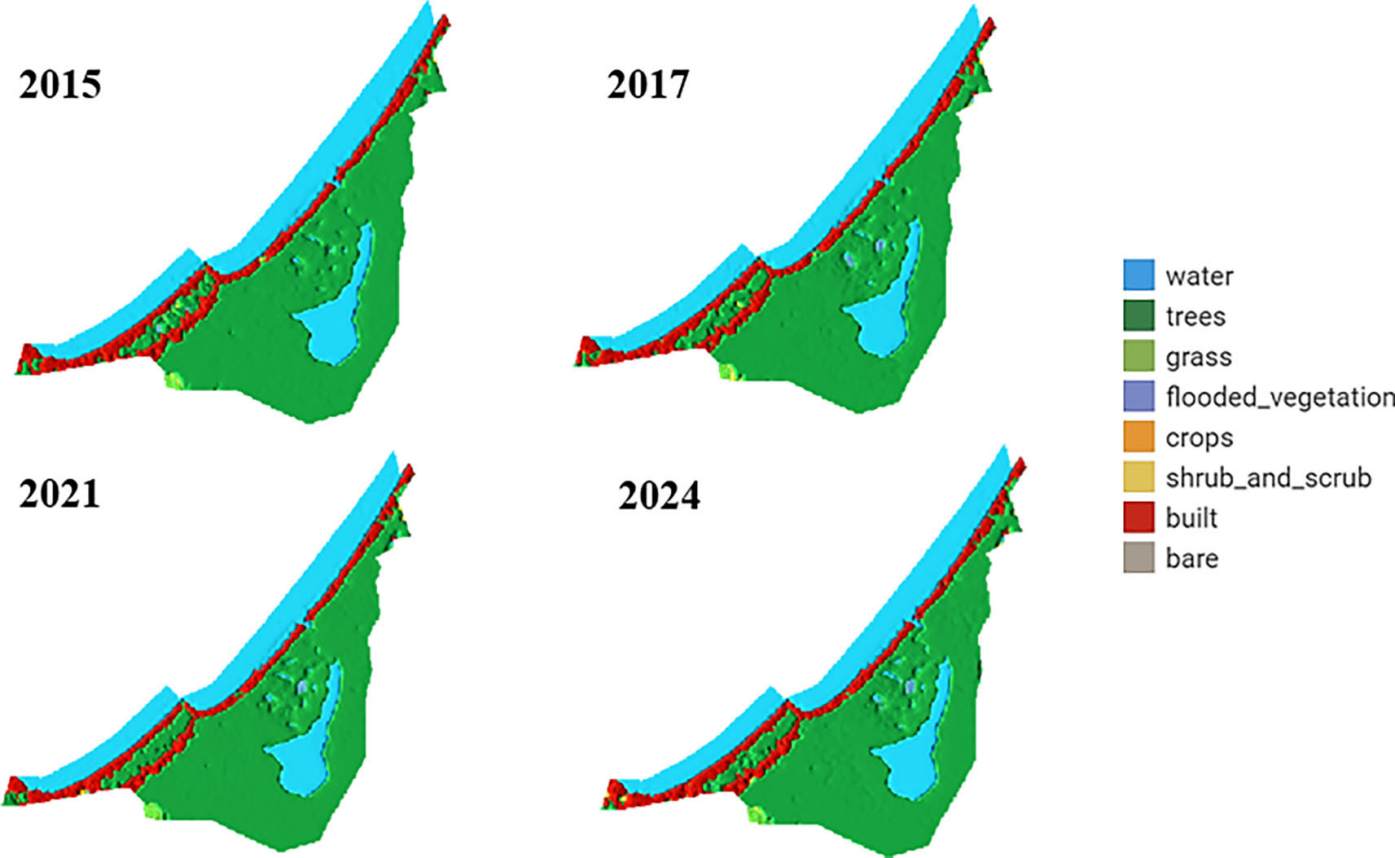
LULC analysis using the Dynamics World platform for the study area.

Figure 6 illustrates LULC analysis using the dynamic world platform for the corresponding study area.

**Figure 7.**
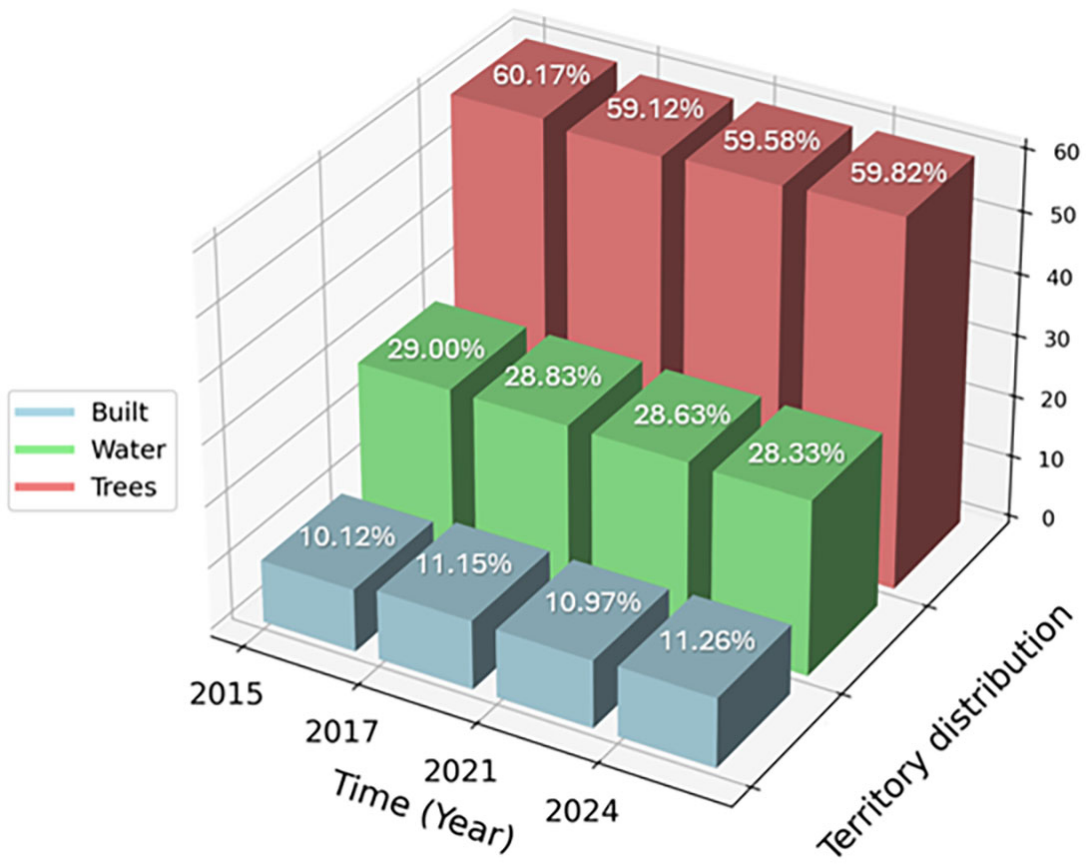
Territory distribution in the La Caimanera marsh for 2015, 2017, 2021 and 2024.

The analysis of LULC images of the La Caimanera marsh, performed from images captured by the Dynamics World platform during the summers of 2015, 2017, 2021, and 2024, revealed remarkable ecosystem resilience. Mangrove cover, predominant in the ROI, showed a slight expansion between 2015 and 2017 and remained stable in the following years, suggesting effective conservation management. In parallel, a subtle initial reduction in the marsh water body was observed, followed by stabilization, possibly attributable to climatic variations or adjustments in local water management. Urban dynamics in the coastal zone exhibited a pattern of controlled growth, as evidenced by a modest expansion of built-up areas between 2015 and 2017, which subsequently stabilized. This phenomenon, together with the absence of predominant agricultural areas, indicates a balance between human development and preservation of the natural ecosystem. Minor fluctuations in grass and shrub cover suggest a dynamic interaction between the natural processes of ecological succession and anthropogenic changes in land-use. Together, these patterns point to integrated and sustainable management of the coastal landscape, where mangrove conservation and urban growth regulation coexist harmoniously, promoting ecosystem resilience in the face of environmental and anthropogenic pressures. The La Caimanera marsh ecosystem shows remarkable stability over the period 2015-2024 with subtle but significant changes in the distribution of its main landscape components, as can be seen in the distribution of the territory in the La Caimanera marsh for 2015, 2017, 2021, and 2024, as illustrated in Figure 7.

Tree cover, predominantly composed of mangroves, has experienced a slight decrease from 60.17% in 2015 to 59.82% in 2024, reflecting a marginal but persistent loss of vegetation. In parallel, the area covered by water showed a similar trend, decreasing from 29% in 2015 to 28.33% in 2024, corroborating the visual observation of a subtle contraction of the marsh water body. In contrast, built-up areas exhibit a gradual increase, rising from 10.12% in 2015 to 11.26% in 2024. This 1.143% increase in built-up areas, although moderate, indicates increasing pressure from human development in the natural ecosystem. The relative stabilization of these percentages between 2021 and 2024 suggests that land management policies may be effective in balancing development demands with conservation imperatives. These quantitative data reinforce the initial interpretation of a resilient ecosystem under careful management, where gradual changes in land cover reflect a delicate balance between mangrove conservation, marsh-water dynamics, and controlled urban growth. The persistence of a high proportion of tree and aquatic cover, despite developmental pressures, underscores the importance and effectiveness of conservation strategies implemented in the region.

### Mangrove cover in the La Caimanera marsh

**Figure 8.**
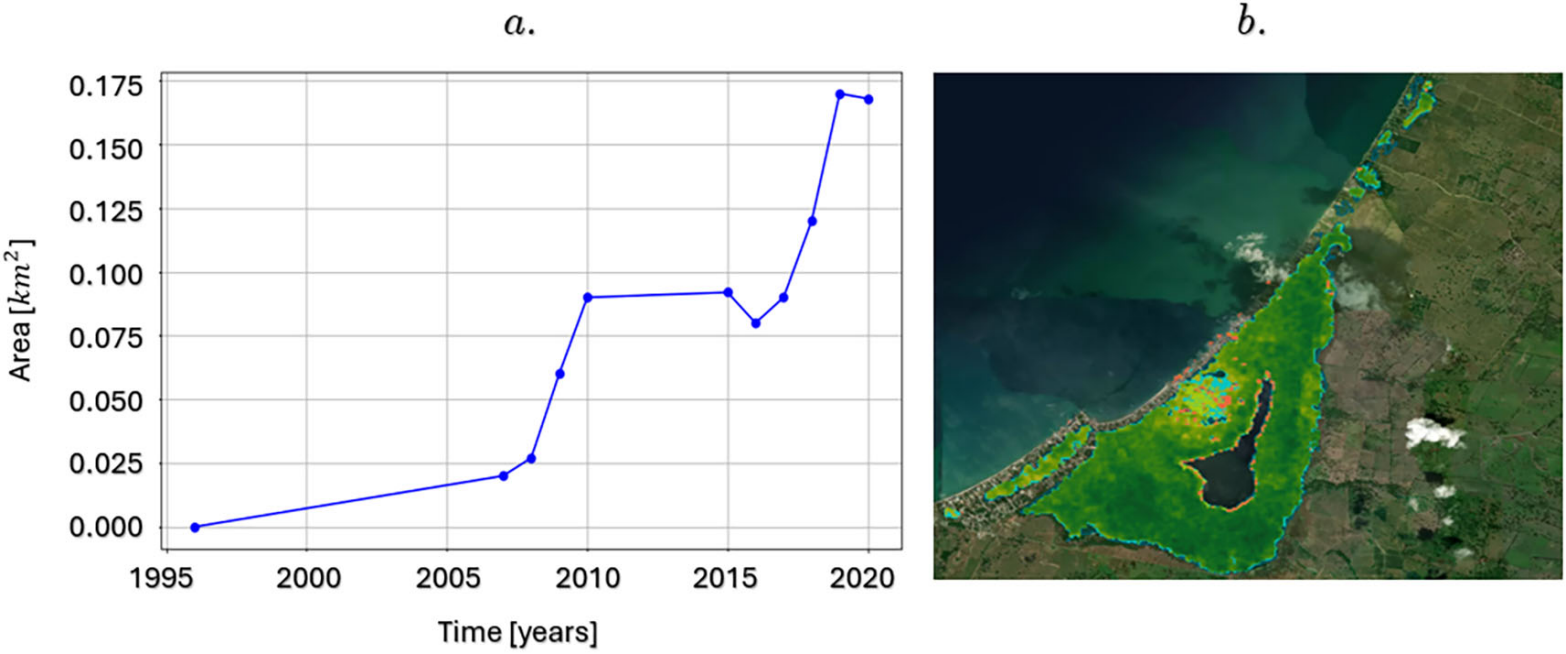
a. Variations in mangrove area in the La Caimanera marsh between 1996 and 2020; b. Distribution of mangrove covers losses and gains.

According to Bunting ^2^ and data obtained through the Global Mangrove Watch platform developed by the Japan Aerospace Exploration Agency (JAXA) and Aberystwyth University, the total area of mangrove habitat in the La Caimanera marsh in 2020 was 12.24 km ^2^, with a coastal linear cover of 7.55 km. Figure 8a shows the changes in mangrove cover, highlighting an increase of approximately 0.17 km ^2^ between 1996 and 2020, equivalent to a 1.3% increase in the total area. It is important to note that, as of 2016, a significant acceleration in this gain of mangrove area was observed, which evidences the joint efforts of the community and environmental management entities in the conservation and restoration of this fundamental ecosystem in the Gulf of Morrosquillo region.

This growth in mangrove cover underscores the success of the protection, restoration, and sustainable management initiatives that have been implemented in the area. Mangroves are not only vital for local biodiversity but also play a key role in climate change mitigation, coastal protection, and the livelihoods of local communities. The acceleration in the recovery of these areas since 2016 suggests greater effectiveness of conservation strategies, which could be replicable in other mangrove ecosystems globally, requiring intervention and recovery mechanisms in density.

**Figure 9.**
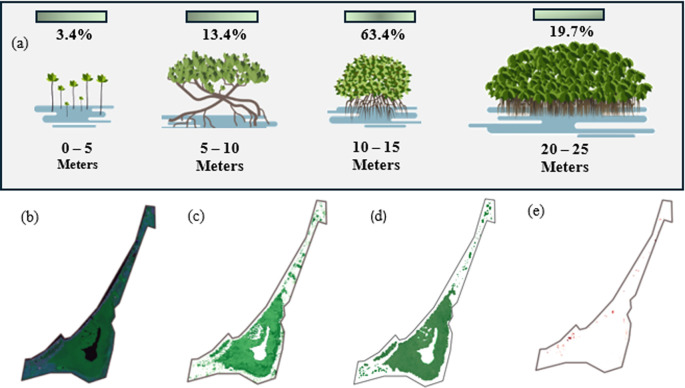
a. Mangrove height classification in La Caimanera Marsh; b. RGB image Mangrove area in 1997; c. Mangrove classifications of 1997; d. Mangrove area 2021; e. Mangrove gain between 1997 and 2021.

Regarding the distribution of mangrove biomass areas in the swamp, that is, the amount of living plant material (leaves, branches, trunks) present per unit area, expressed in tons per hectare (t/h), in 2020 the average amount of aerial biomass was 61.66 (t/h). Detailed percentage distribution of aboveground biomass within different ranges: 31.78% of the mangrove area has a biomass between 0 t/h and 50 t/h, which indicates that a third of the mangrove is young or is being affected by disturbances or is in recovery. Approximately 58.41% of the area has a biomass between 50 and 100 t/h, which is the predominant category, suggesting that most mangroves are composed of healthy adult trees. Likewise, 9.81% had between 100 t/h and 150 t/h, that is, a mature forest with healthy adult trees. Figure 8b shows the recovery of mangrove areas in the La Caimanera marsh over the last 20 years (represented by blue dots) as well as the mangrove areas that have been lost over the same period (represented by red dots). This information is consistent with the minimal changes in land use observed in the data in Figure 6 over the last 10 years. On the other hand, the classification of mangroves according to their height is important for determining the structure and state of the mangrove ecosystem in that area. Using JAXA satellite images, it was found that the maximum canopy height was 12.4 meters, and that the distribution of the entire mangrove area, in terms of height, was established, as shown in Figure 9a. Only 3.4% of the mangrove vegetation in this region consists of trees with heights between 0 and 5 m. This indicates that a very small portion of the mangroves are young or of low stature, which may reflect recent regeneration or areas where mangrove growth is limited, possibly because of salinity and water depth. The highest concentration of mangroves in the area is 10–25 m in height, which is more than 83% of all vegetation. Therefore, the mangrove ecosystem in this area is dominated by mature, well-established trees. This is a sign of a healthy and mature ecosystem in which trees have reached an optimal growth stage. However, it is not without concern that almost one-fifth of the mangroves (19.7 %) are trees that exceed 20 m in height, representing very tall and possibly older trees, which may be key to the overall stability and structure of the ecosystem but also represent the end of the life cycle and should be considered for early renewal.

The multitemporal analysis of the mangrove ecosystem in the La Caimanera marsh was based on a comparison of processed satellite images. The base image in RGB composition ( Figure 9b) clearly distinguishes the two predominant coverages: water bodies and mangrove forests. The application of the binary overlay analysis method between the mangrove classifications of 1997 ( Figure 9c) and 2021 ( Figure 9d) revealed a significant transformation of the ecosystem during this period. The spatial distribution of mangroves in 1997 exhibited a remarkably fragmented pattern, characterized by a discontinuous canopy arrangement, particularly evident in the peripheral areas. In contrast, the 2021 image shows a significantly denser and more homogeneous mangrove cover with a closed and continuous canopy, indicative of a more robust and mature forest structure.

The positive evolution observed during these 24 years suggests effective regeneration of the ecosystem, attributable to multiple converging factors: the successful implementation of conservation measures, the remarkable natural resilience of the system, and an apparent reduction in anthropogenic pressures. This transformation was particularly significant in the central and southern sectors of the study area, where previously fragmented spaces have evolved into continuous and vigorous forest cover. The results obtained not only show a substantial improvement in ecosystem health but also position the La Caimanera marsh as a relevant case study for mangrove restoration and conservation initiatives at regional and global levels, especially considering the increasing pressures that these coastal ecosystems are currently facing. Figure 9e corresponds to the mangrove losses that have occurred in a different time window from 2021 to 2023. It is noticeable that the amount of vegetation cover lost is small, but it has come to interrupt the good growth process that, for the previous time window, 1997 to 2021, showed the mangrove area of the La Caimanera marsh. Similarly, an analysis of changes in mangrove cover in recent years, depicted in Figure 9e, reveals a contrasting trend for the period 2021-2023. Although cover losses during this interval are quantitatively smaller, their occurrence interrupts the pattern of recovery and significant expansion that characterized the previous period (1997-2021). This inflection in the dynamics of vegetation cover, although moderate in extent, is particularly relevant because it marks a break in the positive trajectory experienced by the La Caimanera marsh ecosystem. This change in trend suggests the possible emergence of new pressures on the ecosystem or the intensification of existing stress factors, which merits special attention from the perspective of management and conservation of the area.

**Figure 10.**
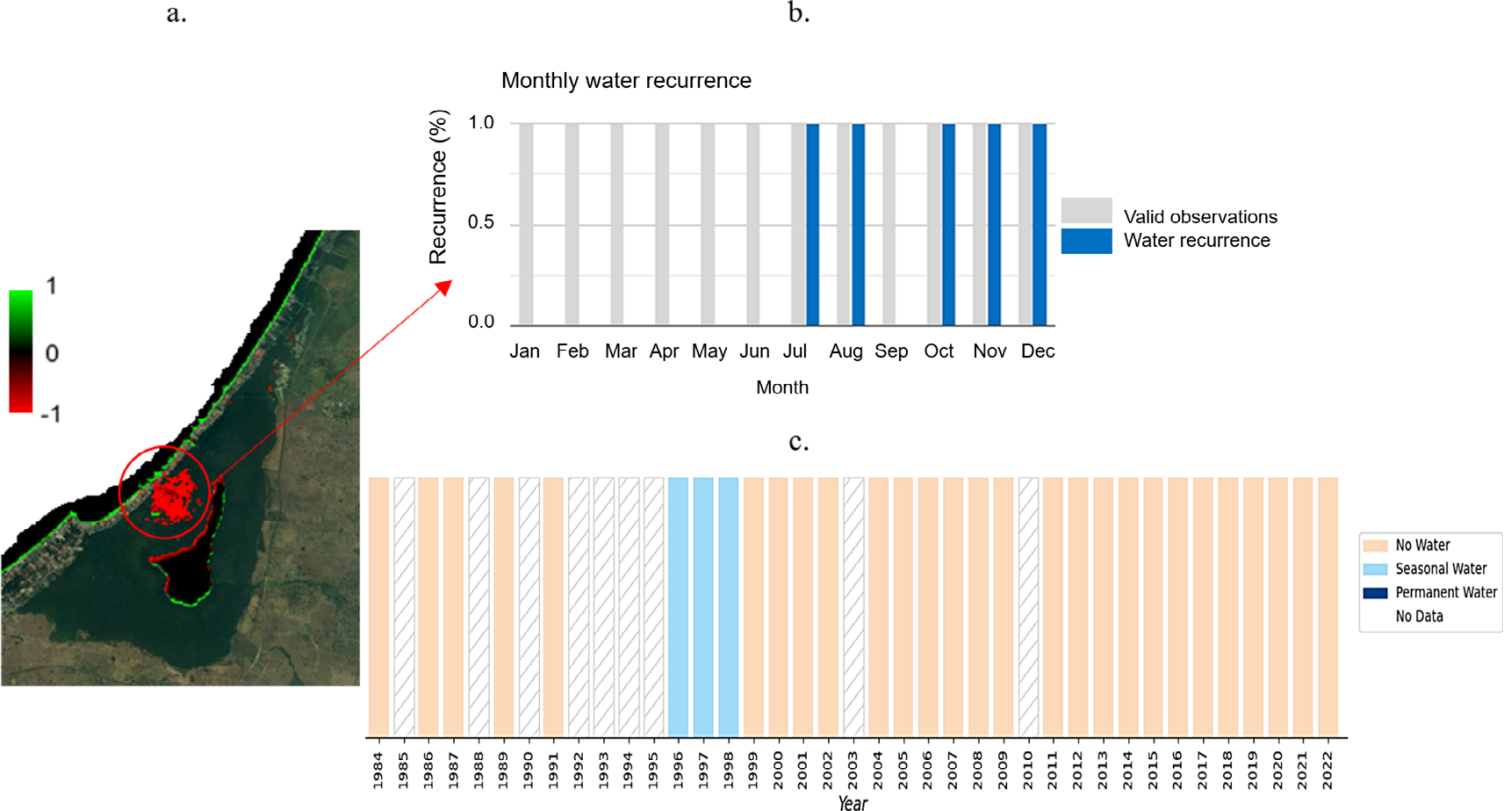
a. Mapping of surface water history in La Caimanera Marsh; b. Monthly water recurrence in marsh; c. Water history in the study area.

Figure 10a presents an analysis of the changes in surface water bodies in the La Caimanera marsh based on annually updated data provided by the application developed by Global Surface Water ( https://global-surface-water.appspot.com/). ^25^ This tool uses over 32 million Landsat and Copernicus satellite images, with a spatial resolution of 30 m, to quantify the variation in global surface water cover. In the analysis, increases in the presence of water are represented in green, whereas decreases are shown in red. A significant change was highlighted in the water masses of the upper sector of the swamp, specifically in the open-water zone located in the center. These changes have been observed between 1984 and 2021. The black areas indicate zones where no significant change in the presence of water was recorded during the same period, while the intensity of the color indicates the degree of change in percentage terms.

Using this application to specifically examine the temporal evolution of surface water presence in the loss area ( Figure 10b, red circle), it was possible to visualize temporal profiles at the pixel level. These profiles showed monthly recurrence over 37 years ( Figure 10b), as well as a history of water presence sorted by seasonality and year over the same period ( Figure 10c). Overall, the plots indicate that at this location (latitude 9.422947°, longitude -75.6332587°), there were no valid observations for 1985, 1988, 1990, 1992-1995, 2003, and 2010 (represented as gaps in the lower plot). Between 1996 and 1999, a high water level was observed, which decreased markedly after this time window. Throughout the 37 years of observation, no water was detected between January and June or September; the months with recurrent water presence were July, August, October, and November ( Figure 10b), in agreement with the rainy season in the region.

## Conclusions

The impact of vegetation cover variation on pollution levels in the La Caimanera marsh (Colombia) was analyzed using remote sensing and imagery. From these findings, it can be inferred that the La Caimanera marsh ecosystem was stable between 2015 and 2024, with a slight reduction in the coverage of mangroves and water bodies, and a slight increase in urbanized areas in the latter part of this time window. These changes reflect a balance between conservation and development, highlighting the effectiveness of land management policies implemented to maintain the ecosystem’s resilience to human pressure. After the evaluation of the NDWI, MNDWI, OC3, and QAA spectral indices calculated in this study, it was possible to accurately characterize the water dynamics and water quality in the La Caimanera marsh. NDWI and MNDWI reflect stability in moisture conditions and water volume between 2010 and 2020, with a slight reduction in water content observed for 2023, which could be related to changes in water quality or quantity. The OC3 index revealed an increase in the chlorophyll-a concentration in 2023 compared to that in 2020, indicating alterations in the biological composition of the water. Likewise, analysis of the QAA index suggests a reduction in turbidity and sediment concentration in the marsh in 2023, a positive sign for the quality of this aquatic ecosystem. These results highlight the relevance of the indices applied to continuously and accurately monitor hydrological and water quality changes in coastal environments. Similarly, the analysis of mangrove height distribution in the area highlights an ecosystem dominated by mature trees, which reflects the health and stability of the natural environment. This predominance, which represents more than 83% of the vegetation, suggests that an ecosystem has reached an ecological balance with adequate conditions for the optimal development of these trees. However, the presence of 19.7% of trees exceeding 20 m highlights a subpopulation of possibly older specimens, which may offer opportunities for future studies focusing on the longevity, adaptability, and resilience of these trees in an evolving ecosystem. In terms of mangrove cover, a significant change towards greater density and homogeneity in its distribution, characterized by a closed and continuous canopy, was observed. This change suggests that the ecosystem has developed a more robust and mature structure, reflecting environmental conditions that favor vigorous growth and stabilization of mangroves. This evolution towards a more uniform and closed canopy is indicative of a positive response of the ecosystem to environmental factors and could be an indication of recovery or resilience to past changes.

## Data Availability

The data and code is availble at: https://github.com/ingccohen/LULC and archived at https://doi.org/10.6084/m9.figshare.29073446. License: CC0 1.0 Universal.
