# Supplementary material for: Analysis of seasonal changes in vegetation cover in the La Caimanera marsh (Colombia) using remote sensing and satellite sensor imagery
Source: F1000Res. 2026 Jan 2;14:748. Originally published 2025 Jul 30. [Version 2] doi: 10.12688/f1000research.164770.2 (PMC12828256; doi:10.12688/f1000research.164770.2)
Supplement: Peer review history

# Reviewer report: Reviewer response for version 2

**DOI:** 10.5256/f1000research.192019.r446747

**Published:** 2026-02-01

**Authors:** Shakila Merchant

**Affiliations:** 1 CUNY Graduate Center, New York, USA; 2 Earth System Environmental Engineering, Grove School of Engineering, City College of CUNY, NY, USA, CUNY Remote Sensing Earth Systems Institute, The City College of CUNY, New York, NY, USA., New York, USA

The revisions looks good and am okay with the edits and revised title. 

 The paper is approved without any reservations or further revisions.

Is the work clearly and accurately presented and does it cite the current literature?

Yes

If applicable, is the statistical analysis and its interpretation appropriate?

I cannot comment. A qualified statistician is required.

Are all the source data underlying the results available to ensure full reproducibility?

Partly

Is the study design appropriate and is the work technically sound?

Yes

Are the conclusions drawn adequately supported by the results?

Yes

Are sufficient details of methods and analysis provided to allow replication by others?

Partly

Reviewer Expertise:

My research explores the dynamic interactions between ecosystems and human systems, with a focus on how natural processes and human-driven impacts—such as climate change, pollution, and urbanization—shape ecological health and resilience. I investigate how these environmental shifts affect both ecosystems and human well-being, particularly in vulnerable and urban communities. Grounded in ecology, my work emphasizes applied, science-based strategies, including nature-based solutions, to promote sustainability, adaptation, and resilience in the face of global and local environmental challenges.

I confirm that I have read this submission and believe that I have an appropriate level of expertise to confirm that it is of an acceptable scientific standard.

# Reviewer report: Reviewer response for version 2

**DOI:** 10.5256/f1000research.192019.r447698

**Published:** 2026-01-22

**Authors:** Roberto Patar Pasaribu

**Affiliations:** 1 Karawang Marine and Fisheries Polytechnic, West Java, Indonesia

1. The introduction is too long; it should focus on matters related to the research title.

 2. The results and discussion should include references from previous research.

 3. The conclusion should quantify changes in mangrove cover or other parameters.

 4. A research paper should clearly link the title, objectives, and conclusions. The paper does not clearly demonstrate the relationship between these three components.

Is the work clearly and accurately presented and does it cite the current literature?

Yes

If applicable, is the statistical analysis and its interpretation appropriate?

Partly

Are all the source data underlying the results available to ensure full reproducibility?

No

Is the study design appropriate and is the work technically sound?

Yes

Are the conclusions drawn adequately supported by the results?

Partly

Are sufficient details of methods and analysis provided to allow replication by others?

Partly

Reviewer Expertise:

Physical Oceanography-(Ocean wave & circulation modeling, Ocean environment modeling for fishery, Oceanic internal waves); Ocean Energy; Marine Engineering; Coastal Hazards; Wave and Currents Modeling; Wind Energy ; Marine and Coastal Management; Marine Environment; Ecology; Marine Science and Environmental Science; Geophysics; Remote Sensing;

I confirm that I have read this submission and believe that I have an appropriate level of expertise to confirm that it is of an acceptable scientific standard, however I have significant reservations, as outlined above.

# Reviewer report: Reviewer response for version 2

**DOI:** 10.5256/f1000research.192019.r446748

**Published:** 2026-01-07

**Authors:** Ronggo Sadono, Adhe Viana Yulida Putri

**Affiliations:** 1 Universitas Gadjah Mada Fakultas Kehutanan, Yogyakarta, Special Region of Yogyakarta, Indonesia

To broaden your search results, use phrases in keywords other than those already in the title.

 Correct the writing of geographical coordinates, such as 9°25'57.42''.

 Improve self-explanatory style, particularly in narrating figures.

Is the work clearly and accurately presented and does it cite the current literature?

Yes

If applicable, is the statistical analysis and its interpretation appropriate?

Yes

Are all the source data underlying the results available to ensure full reproducibility?

Yes

Is the study design appropriate and is the work technically sound?

Yes

Are the conclusions drawn adequately supported by the results?

Yes

Are sufficient details of methods and analysis provided to allow replication by others?

Partly

Reviewer Expertise:

Forest biometrics, modeling, and forest assessment

We confirm that we have read this submission and believe that we have an appropriate level of expertise to confirm that it is of an acceptable scientific standard.

# Reviewer report: Reviewer response for version 1

**DOI:** 10.5256/f1000research.181341.r402027

**Published:** 2025-09-08

**Authors:** Shakila Merchant

**Affiliations:** 1 CUNY Graduate Center, New York, USA; 2 Earth System Environmental Engineering, Grove School of Engineering, City College of CUNY, NY, USA, CUNY Remote Sensing Earth Systems Institute, The City College of CUNY, New York, NY, USA., New York, USA

The topic of research is very interesting and important. The abstract and the introduction can be refined to emphasize this importance. There are many abbreviations within the abstract, which must be spelled out. Many readers may or may not know what MNDWI, or OCS or QAA mean. Also the research speaks about importance of mangrove ecosystem impact on the pollution, however, the pollutants are not depicted - are these heavy metals, air, water pollution, excess nutrients - would help in understanding the impact/influence and thus in creating better/holistic/inclusive management policies that can be effectively implemented. 

 There are links to the data products, however, some seems to be only in non-English language, it would help to make it multi-lingual for easy replicability and use/reference by other potential researchers.

 More details and step-by-step reference on how the download the satellite data and its source were obtained can be provided. Also guidance/ easy to use manual on how to download them for easy use and replicability for other mangrove or marshes would be helpful. 

 Figure 1 can be elaborated further with more details on the images, steps and better captioning would be helpful. 

 A reference to the Random Forest Model would be helpful. 

 More detailed elaboration on how NDVI and LST values were calculated and rationale behind it, which year(s) was it done, in this paragraph would help readers better comprehend the methodologies adopted.. 

 Figure 2 should be at the beginning of the article (introduction rather than results and discussions) and before methodologies. 

 The overall discussion and conclusions emphasizes the mangrove ecosystem's health and services, however, it does not corelate to any pollution/pollutants or impacts of any sort, seems like the title of the research papers is not doing justice to the actual research conducted and results presented here, which is very important. It would rather make sense to change the title to actually emphasize on how this regions' mangrove ecosystem performed over period of time, and how it changed/will change given different water systems, ecosystem/environmental changes due to natural, anthropogenic disasters. This will allow designing better management practices. Alternatively, some linkage to the pollutants (and data if available for same years as this research was done) in the region can also be included.

Is the work clearly and accurately presented and does it cite the current literature?

Yes

If applicable, is the statistical analysis and its interpretation appropriate?

I cannot comment. A qualified statistician is required.

Are all the source data underlying the results available to ensure full reproducibility?

Partly

Is the study design appropriate and is the work technically sound?

Yes

Are the conclusions drawn adequately supported by the results?

Yes

Are sufficient details of methods and analysis provided to allow replication by others?

Partly

Reviewer Expertise:

My research explores the dynamic interactions between ecosystems and human systems, with a focus on how natural processes and human-driven impacts—such as climate change, pollution, and urbanization—shape ecological health and resilience. I investigate how these environmental shifts affect both ecosystems and human well-being, particularly in vulnerable and urban communities. Grounded in ecology, my work emphasizes applied, science-based strategies, including nature-based solutions, to promote sustainability, adaptation, and resilience in the face of global and local environmental challenges.

I confirm that I have read this submission and believe that I have an appropriate level of expertise to confirm that it is of an acceptable scientific standard, however I have significant reservations, as outlined above.

# Reviewer report: Reviewer response for version 1

**DOI:** 10.5256/f1000research.181341.r405047

**Published:** 2025-09-04

**Authors:** Ronggo Sadono, Adhe Viana Yulida Putri

**Affiliations:** 1 Universitas Gadjah Mada Fakultas Kehutanan, Yogyakarta, Special Region of Yogyakarta, Indonesia

This study discusses an interesting topic regarding the dynamics of mangrove forest cover. However, the introduction section does not sufficiently explain the importance of this study. In addition, the methods section lacks detail. It would be beneficial to include a clear and coherent research flow chart. This research can contribute to the development of tools for monitoring changes in land cover in mangrove forests.

 Comments:

**1. Abstract: **Please describe the methodology utilized in the study.

**2. Introduction:**

**-  **The introduction in paragraph 3 is very long and should be divided into two paragraphs.

**- **discusses too much about previous studies but does not go into enough detail to explain the crucial reasons for performing this research.

**3. Study area: **Please also include environmental and social demographic data. This information might offer the reader with an overview of the research site. Furthermore, information about environmental and social characteristics can support the results of this research.

**4. Source of data: **Explain each of the uses for the data sources used.

**5. Result and discussion: **Figure 2: Provide a legend on the map to help readers understand.

Is the work clearly and accurately presented and does it cite the current literature?

Yes

If applicable, is the statistical analysis and its interpretation appropriate?

Yes

Are all the source data underlying the results available to ensure full reproducibility?

Yes

Is the study design appropriate and is the work technically sound?

Yes

Are the conclusions drawn adequately supported by the results?

Yes

Are sufficient details of methods and analysis provided to allow replication by others?

Partly

Reviewer Expertise:

Forest biometrics, modeling, and forest assessment

We confirm that we have read this submission and believe that we have an appropriate level of expertise to confirm that it is of an acceptable scientific standard, however we have significant reservations, as outlined above.
